# The Mediating Effect of Inflammation between the Dietary and Health-Related Behaviors and Metabolic Syndrome in Adolescence

**DOI:** 10.3390/nu14112339

**Published:** 2022-06-02

**Authors:** Ui-Jeong Kim, Eun-Jeong Choi, Hyunjin Park, Hye-Ah Lee, Bomi Park, Haesoon Kim, Youngsun Hong, Seungyoun Jung, Hyesook Park

**Affiliations:** 1 Department of Preventive Medicine, Graduate Program in System Health Science and Engineering, Ewha Womans University, Seoul 07804, Korea; its0912@naver.com (U.-J.K.); hyunjin9191@ewhain.net (H.P.); 2 Department of Preventive Medicine, College of Medicine, Ewha Womans University, Seoul 07804, Korea; choiej_89@naver.com; 3 Clinical Trial Center, Mokdong Hospital, Ewha Womans University, Seoul 07985, Korea; khyeah@ewha.ac.kr; 4 Department of Preventive Medicine, College of Medicine, Chung-Ang University, Seoul 06974, Korea; bomi.s.park@gmail.com; 5 Department of Pediatrics, College of Medicine, Ewha Womans University, Seoul 07804, Korea; hyesk@ewha.ac.kr; 6 Department of Internal Medicine, College of Medicine, Ewha Womans University, Seoul 07804, Korea; imhys@ewha.ac.kr; 7 Department of Nutritional Science and Food Management, Graduate Program in System Health Science and Engineering, Ewha Womans University, Seoul 03760, Korea; sjung131@ewha.ac.kr

**Keywords:** latent class analysis, mediation analysis, metabolic syndrome, health-related behavior, cohort

## Abstract

Chronic diseases develop via complex pathways, depending on the degree of exposure to risk factors from early in life and childhood onward. Metabolic syndrome has multiple risk factors, including genetic factors, inappropriate diet, and insufficient physical activity. This study classified health-related behavior classes in childhood and adolescents and analyzed the direct and indirect effects of each class on the metabolic risk in inflammation-mediated pathways. We identified the health-related lifestyle classes based on health-related behavior indicators in subjects aged 3–15 years who participated in the Ewha Birth and Growth Cohort Study by using a latent class analysis. A mediation analysis was performed to access the direct and indirect effects of each class on the continuous metabolic syndrome score (cMetS), with the inflammatory index used as a mediating factor. Subjects were classified into inactive and positive lifestyle classes according to their characteristics. In the inactive lifestyle class, interleukin (IL)-6 and cMetS had a significant association. The study confirmed that IL-6 exerts a significant indirect effect between inactive lifestyle and cMetS. This result supports previous studies. Since the health behaviors of children and adolescents can affect the likelihood of subsequent metabolic syndrome, appropriate health behavior interventions for this period are needed.

## 1. Introduction

Metabolic syndrome is diagnosed when a patient has three or more of the following: abdominal obesity, high blood pressure, high fasting glucose level, hypertriglycemia, or low high-density lipoprotein cholesterol (HDL-c) levels [1]. It increases the risks of death from cardiovascular disease, type 2 diabetes, and stroke [2]. The worldwide prevalence of metabolic syndrome is 20–25% in adults [3,4] and 19.2% in children [5]. In Korea, one in five adults (22.9%) had metabolic syndrome in 2018 [6].

Chronic disease develops via complex pathways, depending on the degree of exposure to risk factors from early in life and childhood onward. The World Health Organization (WHO) states that a “life-course approach” should be taken to prevent chronic diseases [7]. Metabolic syndrome has multiple risk factors, including genetic factors, inappropriate dietary habits, and insufficient physical activity [8,9]. Risk factors related to lifestyle habits accumulated over the time, and many studies have been reported on the relationship between lifestyle and health. A recent study reported a change in life expectancy with the cessation of smoking, consumption of a healthy diet, performance of moderate-intensity exercise for at least 30 min a day, and maintenance of a normal body weight. Cancer patients who implemented healthy habits survived for an average of 22.9 years, which was more than twice as long as those who did not (11 years) [10]. Moreover, some studies reported that low-level chronic inflammation may cause metabolic syndrome [11].

Adolescence is a transitional period from puberty to adulthood and is an important stage in the development and maintenance of health-related behaviors [12]. It has been reported that health-related behaviors and lifestyles learned in early childhood and adolescence are difficult to change, so it can be said that chronic diseases are caused by health behaviors acquired during this period [13]. However, the majority of adolescents are reported to have a lower adherence to healthy lifestyles even when they are critically ill [14]. This low prevalence of healthy lifestyles during adolescence poses a significant public health challenge as it increases future chronic disease risk later in adulthood and lowers the quality of life during adolescence.

Therefore, adolescence can be regarded as an important period for preventive management in which chronic diseases can be prevented through interventions for the formation of healthy habits [15]. This study classified health-related behavior classes in childhood and adolescents and analyzed the direct and indirect effects of each class on metabolic risk in inflammation-mediated pathways.

## 2. Materials and Methods

### 2.1. Participants

This study was conducted on the prospective cohort of the Ewha Birth and Growth Study, which enrolled mothers at 24–28 weeks of pregnancy from 2001 to 2006 at the Department of Obstetrics and Gynecology, Ewha Womans University Mokdong Hospital (Seoul, Korea). Since 2005, 940 children of these women who agreed to participate in the study have been followed annually [16]. At each follow-up examinations, blood and urine samples collection after fasting for at least 8 h, anthropometric measurements, and questionnaires were performed. This study examined 249 subjects aged 13–15 years who participated in at least two follow-up examinations and did not have missing metabolic index data. All participants provided informed consent and the study protocol was approved by the Institutional Review Board of Ewha Womans University Seoul Hospital (IRB number: SEUMC 2020-07-016).

### 2.2. Exposure

Physical activity, sedentary behavior, and dietary habits are important modifiable determinants in adolescents. The health-related behavior classes were based on health-related behavior indicators: subjective health status, physical activity, dietary inflammatory index, and secondhand smoke exposure. These indicators were measured repeatedly at the ages of 3–15 using questionnaires and included in the analysis if observed at least twice.

To measure the subjective health, physical activity, and sedentary lifestyle of children aged from 3 to 15 years during the study follow-up, we used modified questionnaires with reference to the Korea National Health and Nutrition Examination Survey. Subjective health status was determined based on self-reported health status (very good, good, average, bad, or very bad). The performance of vigorous physical activity was determined if participants reported more than three sessions of at least 20 min activities on each occasion that made them out of breath or sweaty over the past week. The daily leisure sedentary lifestyle was estimated based on the time spent on activities, such as watching TV, surfing the internet, and playing games, over the past week. Then, participants were determined as “inactive” if they reported more than three times of daily sedentary leisure greater than two hours over the past week. Shivappa et al. [17] developed a dietary inflammatory index (DII) through a systematic literature review. The index is used to estimate dietary inflammation based on the intakes of 36 nutrients and 9 foods. Calculation of the DII is based on dietary intake that provided a robust estimate of a mean and standard deviation in a world database for each parameter. Dietary data for each participant expressed an individual’s exposure as a Z-score. We get the “standard mean” by subtracting from the actual reported parameter value and divided by its standard deviation. This Z-score was converted to a percentile score to minimize the effect of right-skewing and centered values on 0 and bounded them between −1 (reflect the anti-inflammatory potential of the diet) and +1 (reflect proinflammatory potential of the diet), each percentile score was doubled and then “1” was subtracted. Then, this value was respectively multiplied by the inflammatory effect score of each food to obtain the specific DII score of each parameter. All specific DII scores were then summed for each participant in the study [17]. We investigated 24 food groups using a food frequency questionnaire at the age of 13–15 years and we assessed them based on the method from Shivappa et al. [17]. The urine level of cotinine, a nicotine metabolite, was used as a measure of secondhand smoke. Cotinine was measured using a high-performance liquid chromatography–triple tandem mass detector, HPLC–MS/MS (Agilent 6490b, Agilent, Santa Clara, CA, USA). Urine samples were collected at each follow-up and stored at −80 °C. The limit of detection (LOD) was 0.141 μg/L; any measured value lower than LOD was replaced with LOD/√2.

### 2.3. Mediator

The inflammatory markers high-sensitivity C-reactive protein (hs-CRP) and interleukin (IL)-6 in the adolescents were considered as mediator. hs-CRP was measured using a particle-enhanced immune turbidimetric assay (Cobas 8000 C702 analyzer; Roche, Mannheim, Germany) and IL-6 was measured using a VersaMax microplate reader (Molecular Devices, Sunnyvale, CA, USA). Since the hs-CRP and IL-6 data were not normally distributed, they were converted into log values. The LOD for hs-CRP was 0.15 mg/dL and any measured value lower than LOD was replaced with LOD/√2. The coefficients of variation (CV) of hs-CRP and IL-6 were less than 10% in all measurements.

### 2.4. Outcome

Since the components of metabolic syndrome in adolescence have not been defined [18], continuous metabolic syndrome scores (cMetS) at the age of 13–15 years was calculated using body mass index (BMI), mean arterial pressure (MAP), fasting blood glucose, triglyceride (TG), and high-density lipoprotein-cholesterol (HDL-c) to determine the risk of MetS in adolescence. BMI was used as an index of obesity and calculated as weight (kg) divided by height squared (m^2^). MAP is an index of blood pressure that has a smaller standard deviation than the systolic blood pressure (SBP) and diastolic blood pressure (DBP) and it was calculated as DBP + [(SBP − DBP)/3]. Blood chemistry tests were performed for fasting blood glucose, TG, and HDL-c levels. Each metabolic indicator was standardized using the Z-score method with consideration of sex. To calculated cMetS, Z-scores of each metabolic indicator were summed. Since HDL-c has a reversal causality with cMetS, its Z-score was multiplied by −1 [19,20].

### 2.5. Covariates

Sex, age, and parents’ monthly household income were considered as covariates. The parents’ monthly household income was divided into three categories (less than 3 million won, 3–5 million won, and 5 million won or more). The association between these factors and health-related behaviors has been demonstrated previously [21,22].

### 2.6. Statistical Analysis

For the descriptive statistics, the mean and standard deviation were calculated for normally distributed continuous variables and nonnormally distributed continuous variables are shown as median and interquartile range (IQR). Categorical variables are presented as frequencies with percentages.

We constructed a trajectory model in ages 3 to 15 years to assess the changes in participant’s health-related behaviors using PROC TRAJ in the SAS program (SAS Institute, Cary, NC, USA). We selected the appropriate model based on the group distribution and the Bayesian information criterion (BIC) [23].

Based on the changes in behavior patterns, a latent class analysis (LCA) was performed to identify health-related behavior classes. LCA can distinguish different classes. The main assumption of LCA is that latent class and observational variables are categorical, and each observed variable is independent and conditional on the class. The latent class model estimates the probability of a given individual belonging to each class [24]. Typically, the number of classes in the model produced by LCA is increased one class at a time. Several model-fit indices are used, including the Akaike information criterion (AIC) and BIC. Since the BIC is a commonly used criterion [25], it was prioritized here.

The fitness indexes of the LCA models of patterns of health-related behavior changes were compared. Two concise statistical LCA models with best fits measured by a lower BIC were selected (BIC = 69.99, aBIC = 35.12) (Supplementary Materials Table S1).

To confirm each health-related behavior class and its effect on cMetS, an association analysis was performed, and a mediation analysis was performed considering inflammatory markers as a mediating factor. A mediation analysis is a statistical method for determining causality between input variables and the outcome variable. Causal variables can have direct or indirect (via a mediator) effects on the outcome variable. This study used the Process-Macro proposed by Hayes [26].

All statistical analyses were performed using SAS software (version 9.4; SAS Institute, Cary, NC, USA). In all analyses, *p* values < 0.05 in two-tailed tests were considered statistically significant.

## 3. Results

Table 1 summarizes the characteristics of the subjects. There were 122 males (49.0%) and 127 females (51.0%), with an average age of 13.28 ± 0.59 years. Of the subjects, 58.9% had a “good” subjective health status, and there was no sex difference. Regarding vigorous physical activity, once or twice a week was the most dominant class (38.5%), while that for sedentary leisure time was ≥2 h a day (42.7%). Of the boys and girls, 35.3% and 24.0%, respectively, performed vigorous physical activity at least 3–4 times a week, while 15.6% and 31.2%, respectively, rarely participated in vigorous physical activity. Physical activity levels differed significantly between the sexes, while sedentary leisure time did not. The dietary inflammatory index was positive for girls (0.24 ± 1.95), but not for boys (−0.25 ± 1.72). There was no gender difference in the monthly household income.

**Figure 1:**
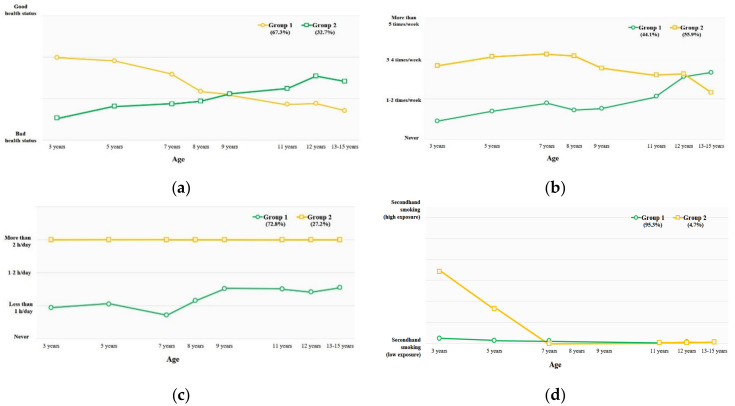
Trajectories of health-related behaviors from 3 to 15 years of age: (**a**, Subjective health status) 1: getting worse health status, 2: getting better health status; (**b,** Vigorous physical activity) 1: increasing physical activity group, 2: slight decreasing physical activity group; (**c,** Sedentary lifestyle) 1: low sedentary lifestyle group, 2: high sedentary lifestyle group; (**d,** Cotinine) 1: low level of cotinine as secondhand smoking in childhood group, 2: high level of cotinine as secondhand smoking in childhood.

Figure 1 shows the changes in health-related behavioral indicators. Regarding subjective health status, the respondents reported that it was getting better (32.7%) and worse (67.3%), respectively. The respondents reported an increase (44.1%) and slight decrease (55.9%), respectively, regarding their vigorous physical activity, while their sedentary leisure levels remained high (27.2%) and low (72.8%), respectively. For secondhand smoke exposure in childhood, high (4.7%) and low (95.3%) exposure groups were distinguished.

**Figure 2:**
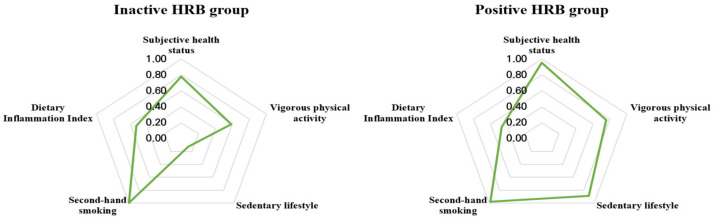
Health-related behavior patterns for each group. HRB, health-related behavior.

Figure 2 shows the conditional probabilities of health-related behaviors. In the figure, the closer each specific health-related behavior factor is to 1.00, the higher the probability that the subjects in each group have a healthy behavior for each specific factor. Class 1 (inactive lifestyle class, *n* = 102) subjects were less likely to engage in vigorous physical activity, and more likely to engage in sedentary leisure activities. Class 2 (positive lifestyle class, *n* = 127) subjects tended to have an overall healthy pattern of behavior. There was no significant difference between the classes in subjective health status, likelihood of having an inflammatory diet, or exposure to secondhand smoke in childhood and childhood.

Table 2 shows the results from the models using a linear regression analysis. In the crude model, the inactive health-related behavior class was significantly associated with greater levels of IL-6 (*β* = 0.187, SE (standard error) = 0.069, *p* = 0.007) and cMetS (*β* = 0.921, SE = 0.392, *p* = 0.020), whereas the positive association between the inactive health-related behavior class and hs-CRP (*β* = 0.228, SE = 0.124) were marginally significant (*p* = 0.067). After adjusting for sex, age, and monthly household income, the positive associations remained significant with IL-6 (*β* = 0.168, SE = 0.072, *p* = 0.02) and marginally significant with cMetS (*β* = 0.751, SE = 0.405, *p* = 0.065). However, hs-CRP was not statistically significantly associated with inactive health-related behavior class.

**Figure 3:**
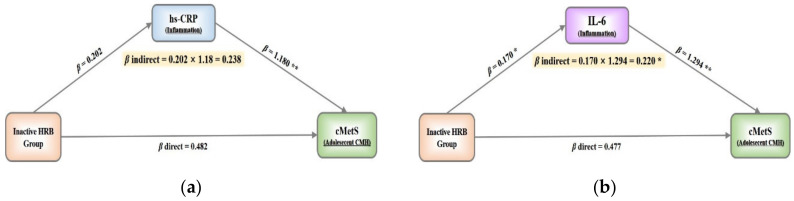
The effect of mediators (M) on the association between health-related behaviors group (X) and continuous metabolic syndrome score (Y): (**a**, hs-CRP) considering hs-CRP as mediators; (**b**, IL-6) considering IL-6 as mediators. * *p* < 0.05, ** *p* < 0.01. HRB, health-related behavior; hs-CRP, high-sensitivity C-reactive protein; IL-6, interleukin-6; cMetS, continuous metabolic syndrome score; CMH, Cardiometabolic health. All models were adjusted by sex, age, and monthly household income.

Mediation analysis was performed to access the direct and indirect effects of each class on the metabolic risk in inflammation-mediated pathways (Figure 3). When hs-CRP was considered as a mediator, we observed a nonsignificant direct effect of the inactive health-related behavior class on cMetS (*β* = 0.482, 95% CI (confidence interval) −0.268 to 1.231). Similarly, there was a suggestion of an indirect effect of the inactive health-related behavior class on cMetS via hs-CRP, but the results did not reach statistical significance (*β* = 0.238, 95% CI −0.065 to 0.580) (Figure 3A). When IL-6 was considered as a mediator, we observed a nonsignificant direct effect of the inactive health-related behavior class on cMetS (*β* = 0.477, 95% CI −0.320 to 1.273). However, we observed a significant indirect effect of the inactive health-related behavior class on cMetS via the IL-6 pathway (*β* = 0.220, 95% CI 0.040 to 0.456). When analyzing the specific IL-6 pathway, an inactive health-related behavior was significantly positively associated with IL-6 (*β* = 0.170, *p* < 0.05) and IL-6 was significantly associated with cMetS (*β* = 1.294, *p* < 0.01). The entire path was “inactive health-related behavior class” –“IL-6” –“cMetS” (Figure 3B).

## 4. Discussion

This study distinguished between inactive lifestyle class and positive lifestyle class based on repeated measures of health-related behaviors. The IL-6 level of the inactive lifestyle class was 0.168 higher than that of the positive lifestyle class, while cMetS was 0.751 higher. Moreover, IL-6 had a significant indirect effect on the relationship between the inactive lifestyle class and cMetS in the mediated pathway (*β* = 0.220, 95% CI 0.040–0.456).

An association between an inactive lifestyle and metabolic syndrome has been reported previously, and the association between negative health behaviors and metabolic syndrome is also well known. In a study examining the effects of health-related behavioral changes on the prevalence of metabolic syndrome in adults over 40 years of age, the prevalence increased by 9% in a persistent heavy drinking compared to persistent moderate drinking group. In addition, the risk of MetS decreased by 30.3% in the “continuous physical activity group” compared to “continuous passive activity group” [27].

Many studies have reported an association between physical activity and metabolic syndrome. In this study, the physical activity indicators were daily leisure time and the frequency of vigorous physical activity; the cMetS was high in the low-physical activity group. A Portuguese study found that a sedentary lifestyle, excessive caloric intake, and obesity tended to increase the incidences of diabetes and metabolic syndrome [28]. Meanwhile, a Swedish population-based cohort study reported that the odds ratio of metabolic disease among those who cited “watching TV” and “low physical activity” in the context of their daily leisure time during adolescence was 2.14 times higher than that of those who were physically active [29].

A study of the eating patterns of 14-year-olds in Western Australia reported that the risk of metabolic disease was about 2.5 times higher in those frequently consuming a Western diet [30]. In a 2018 meta-analysis of cross-sectional and cohort studies, a high dietary fiber intake was inversely proportional to metabolic syndrome in the general population [31,32]. A cohort study reported a significant association of the Dietary Inflammatory Index with metabolic syndrome and its components, even after adjusting for various potential confounders [33]. However, in this study, we found no significant difference in the DII between the inactive and positive lifestyle groups. Given that individuals who participate in cohorts tend to be more health conscious, this result may reflect an overall high quality of diet of our study participants with less variation in dietary inflammation potential. Indeed, the distribution of DII in our study was relatively narrow with a low mean value.

An association between bad eating habits and metabolic syndrome has been reported. The Korean Nurses’ Health Study, which analyzed the risks posed by the unhealthy eating habits associated with the nursing profession, found that consuming ≥50% of one’s daily calories after 7 pm, frequent consumption of carbonated drinks, and irregular or short meals were associated with metabolic syndrome [34].

In the mediated pathway analysis conducted in this study, a significant relationship of IL-6, but not hs-CRP, with the cMetS was observed. The Mater-University of Queensland Study of Pregnancy cohort study demonstrated that negative health behaviors, such as smoking, partially contributed to inflammation [35]. Low-level chronic inflammation may be associated with metabolic dysfunction, which can lead to metabolic syndrome [36]. Although the latter study examined adults, the results were similar to this study, in suggesting that inflammation contributes to the long-term risk of disease.

LCA is a popular method for identifying classes, which uses a maximum likelihood estimation to distinguish internally homogeneous and externally heterogeneous subgroups. The relationships of these subgroups with risk and protective factors can then be investigated [37]. Another advantage of LCA is that it is a model-based approach and is thus more flexible than conventional clustering techniques; it determines the probability that a given individual belongs to a specific class, such that patient selection criteria are less arbitrary [24,38].

This study had several limitations. First, since the subjects were recruited from a single hospital, it is difficult to generalize the results. Second, since inflammatory indicators and metabolic health were both investigated in adolescence, there may have been a bias toward an inverse correlation, i.e., measurement error. Third, since the questionnaire used was self-written, there may have been a reporting bias. Lastly, our study population had relatively high levels of vigorous physical activity (48.7% in boys and 28.5% in girls). Nonetheless, these levels of physical activity were similarly observed in the national data (45.8% in boys and 23.7% in girls from the Korea Youth Risk Behavior Web-based Survey [39]), supporting the representativeness of our data when applied to the Korean population.

Despite its limitations, this study identified latent classes based on changes in health-related behaviors and confirmed a significant indirect effect of inflammatory indicators on the relationships of the changes in health-related behaviors with metabolic syndrome. These results support previous studies. Since the health behaviors of children and adolescents can affect the likelihood of subsequent metabolic syndrome, appropriate health behavior interventions for this period are needed.

## 5. Conclusion

In summary, we classified health-related behavior classes in childhood and adolescents, and analyzed the direct and indirect effects of each class on metabolic risk in inflammation-mediated pathway. The IL-6 level and cMetS of the inactive lifestyle class was higher than that of the positive life-style class. Also, IL-6 had a significant indirect effect on the relationship between the inactive lifestyle class and cMetS in the mediated pathway. This study is meaningful in that it has confirmed a significant effect on the relationship between health-related behavior and cMets in the indirect path considering inflammation indicator and supports the results of previous studies.

## Figures and Tables

**Table 1:** Characteristics of study subjects aged 13–15 years.

	Total (*n* = 249)	Boys (*n* = 122)	Girls (*n* = 127)	*p*-Value
Age (years)	13.28 ± 0.59	13.24 ± 0.53	13.31 ± 0.64	0.301
Subjective health status *	183 (58.9%)	96 (39.0%)	87 (35.4%)	0.223
Vigorous physical activity (more than 20 min)				
Never	58 (23.3%)	19 (15.6%)	39 (31.2%)	0.004
1–2 times/week	95 (38.5%)	45 (36.9%)	50 (40.0%)	
3–4 times/week	73 (29.6%)	43 (35.3%)	30 (24.0%)	
≥5 times/week	21 (8.5%)	15 (12.3%)	6 (4.8%)	
Sedentary lifestyle				
Never	4 (1.63%)	1 (0.8%)	3 (2.4%)	0.988
Less than 1 h/day	64 (26.0%)	31 (26.4%)	33 (26.4%)	
1–2 h/day	73 (29.7%)	36 (29.8%)	37 (29.6%)	
More than 2 h/day	105 (42.7%)	53 (43.8%)	52 (41.6%)	
Dietary Inflammation Index	0.00 ± 1.85	−0.25 ± 1.72	0.24 ± 1.95	0.037
hs-CRP (mg/dL)	0.16 (0.11, 0.38)	0.20 (0.11, 0.48)	0.11 (0.11, 0.33)	0.013
IL-6 (pg/mL)	2.74 (2.02, 3.65)	2.72 (2.01, 3.48)	2.76 (2.08, 3.76)	0.788
cMetS	0.00 ± 3.03	0.00 ± 3.15	0.00 ± 2.92	0.425
Monthly household income, KRW				
<KRW 3 million	16 (6.6%)	7 (5.8%)	9 (7.3%)	0.739
KRW 3–5 million	68 (27.9%)	34 (28.1%)	34 (27.6%)	
≥KRW 5 million	160 (65.6%)	80 (66.1%)	80 (65.0%)	

* Subjective health good status. Values are presented as mean ± SD (standard deviation) or median (interquartile range) or *n* (%). hs-CRP, high-sensitivity C-reactive protein; IL-6, Interleukin-6; cMetS, continuous metabolic syndrome risk score; KRW, Korean won.

**Table 2:** Multiple linear regression analysis for the association between latent group for health-related behaviors and metabolic risk factors.

		hs-CRP			IL-6			cMetS		
		*β*	SE	*p*-Value	*β*	SE	*p*-Value	*β*	SE	*p*-Value
Crude model	Inactive HRB group	0.228	0.124	0.067	0.187	0.069	0.007	0.921	0.392	0.02
	Positive HRB Group									
Adjusted model	Inactive HRB group	0.202	0.129	0.118	0.168	0.072	0.02	0.751	0.405	0.065
	Positive HRB group									

Reference group: positive HRB group. Adjusted model was adjusted by sex, age, and monthly household income. HRB, health-related behavior; SE: standard error.

## Data Availability

The cohort data are not freely available, but the Ewha Birth and Growth Study team welcomes collaborations with other researchers. For further information, contact Hyesook Park (the corresponding author).

## Supplementary Materials

The following are available online at https://www.mdpi.com/article/10.3390/nu14112339/s1, Table S1: The fit index for each latent class model.
