# Supplementary material for: The Mediating Effect of Inflammation between the Dietary and Health-Related Behaviors and Metabolic Syndrome in Adolescence
Source: Nutrients. 2022 Jun 2;14(11):2339. doi: 10.3390/nu14112339 (PMC9182841; doi:10.3390/nu14112339)
Supplement: Supplementary file 1 [file nutrients-14-02339-s001.zip › nutrients-1740240-supplementary.pdf]

Table S1. The fit index for each latent class model.

	**Class 2**	**Class 3**	**Class 4**	**Class 5**
AIC	31.30	39.36	50.97	62.12
BIC	69.99	99.15	131.87	164.13
Adjusted BIC	35.12	45.26	58.96	72.20
Likelihood G^2	9.30	5.36	4.97	4.12
CAIC	80.99	116.15	154.87	193.13

AIC: Akaike Information Criteria; BIC: Bayesian Information Criteria; CAIC: conditional Akaike Information Criteria; Adjusted BIC: Adjusted Bayesian Information Criteria; Likelihood G^2: Likelihood G-squared.
